# Frequency of nutritional disorders and their risk factors among children attending 13 nurseries in São Paulo, Brazil. A cross-sectional study: Frequência de distúrbios nutricionais e de seus fatores de risco entre crianças de 13 creches de São Paulo, Brasil. Um estudo transversal

**DOI:** 10.1590/1516-3180.2014.8800711

**Published:** 2015-08-03

**Authors:** Tulio Konstantyner, José Augusto Aguiar Carrazedo Taddei, Thais Cláudia Roma Oliveira Konstantyner, Laura Cunha Rodrigues

**Affiliations:** I MD, MSc, PhD. Professor, Department of Health Sciences, Universidade de Santo Amaro (Unisa), São Paulo, Brazil.; II MD, MSc, PhD. Affiliated Professor, Department of Pediatrics, Escola Paulista de Medicina-Universidade Federal de São Paulo (EPM-Unifesp), São Paulo, Brazil.; III MSc, PhD. Professor, Department of Epidemiology, Faculdade de Saúde Pública, Universidade de São Paulo (FSP-USP), São Paulo, Brazil.; IV MD, MSc, PhD. Head of Faculty of Epidemiology and Population Health, London School of Hygiene and Tropical Medicine, London, United Kingdom.

**Keywords:** Risk factors, Child day care centers, Nutritional status, Infant, Health promotion, Fatores de risco, Creches, Estado nutricional, Lactente, Promoção da saúde

## Abstract

**CONTEXT AND OBJECTIVE:**

Nutritional disorders are associated with health problems earlier in life. The objective here was to estimate the frequency of nutritional disorders and their risk factors among children.

**DESIGN AND SETTING:**

Cross-sectional study in nurseries at 13 day-care centers in São Paulo, Brazil.

**METHODS:**

The mothers of 482 children were interviewed, with anthropometry on these children. Children whose anthropometric indices for weight and height were greater than two standard deviations were considered to have nutritional disorders.

**RESULTS:**

Children in families with lower *per capita* income (odds ratio [OR]: 2.25; 95% confidence interval, CI: 1.08-4.67) and who presented neonatal risk (OR 8.08; 95% CI: 2.29-28.74), had incomplete vaccinations (OR 3.44; 95% CI: 1.15-10.31) or were male (OR 3.73; 95% CI: 1.63-8.56) were more likely to be malnourished. Children in families with lower *per capita* income were also less likely to be overnourished (OR 0.40; 95% CI: 0.19-0.88). Children who were exclusively breastfed for less than two months (OR 2.95; 95% CI: 1.35-6.44) or who were male (OR 2.18; 95% CI: 1.02-4.65) were also at greater risk of being overnourished. Children who presented neonatal risk (OR 3.41; 95% CI: 1.04-11.23), had incomplete vaccinations (OR 3.18; 95% CI: 1.30-7.76), or were male (OR 2.76; 95% CI: 1.56-4.90) were more likely to have a nutritional disorder.

**CONCLUSIONS:**

Nutritional disorders remain present in children attending nurseries in São Paulo. Actions should focus on boys, children who were exclusively breastfed for less than two months and those without up-to-date vaccinations.

## RESUMO

**CONTEXTO E OBJETIVO:**

Distúrbios nutricionais estão associados a problemas de saúde mais cedo na vida. O objetivo foi estimar a frequência dos distúrbios nutricionais e seus fatores de risco entre crianças.

**DESENHO E LOCAL:**

Estudo transversal em berçários de 13 creches em São Paulo, Brasil.

**MÉTODOS:**

Foram realizadas entrevistas com as mães e antropometria de 482 crianças. Foram consideradas com distúrbios nutricionais as crianças com valores dos índices antropométricos para peso e estatura maiores do que dois desvios-padrão.

**RESULTADOS:**

Crianças de famílias com menor renda *per capita* (OR 2,25; intervalo de confiança de 95%, IC 95%: 1,08-4,67), de risco neonatal (OR 8,08; IC 95%: 2,29-28,74), com vacinação incompleta (OR 3,44; IC 95%: 1,15-10,31) ou do sexo masculino (OR 3,73; IC 95%: 1,63-8,56) apresentaram maior risco de estarem desnutridas. Crianças de famílias com menor renda *per capita* apresentaram menor risco de estarem hipernutridas (OR 0,40; IC 95%: 0,19-0,88) e crianças que receberam amamentação exclusiva por menos de dois meses (OR 2,95; IC 95%: 1,35-6,44) ou do sexo masculino (OR 2,18; IC 95%: 1,02-4,65) apresentaram maior risco de estarem hipernutridas. Crianças de risco neonatal (OR 3,41; IC 95%: 1,04-11,23), com vacinação incompleta (OR 3,18; IC 95%: 1,30-7,76), ou do sexo masculino (OR 2,76; IC 95%: 1,56-4,90) apresentaram maior risco de terem um distúrbio nutricional.

**CONCLUSÕES:**

Distúrbios nutricionais permanecem presentes em crianças frequentadoras de creches em São Paulo. Ações devem focar meninos, crianças que foram amamentadas exclusivamente por menos de dois meses e aquelas com vacinação incompleta.

## INTRODUCTION

Nutritional disorders caused by a nutritional imbalance, consisting of either malnutrition or overnutrition, are associated with serious health problems and the risk of premature illness and earlier death.^1^^,^^2^ Both factors have been used as indicators of child health status, since children’s health has multifactorial determinants, including socioeconomic status, food consumption, healthcare conditions, infectious diseases and biological factors.^3^^,^^4^

During the first years of life, anthropometric measurements, i.e. length/height, weight and/or age, that are two standard deviations (SDs) below (malnutrition) or two SDs above (overnutrition) the mean value for the reference (healthy) population have been correlated with negative effects on health, development and behavior, with subsequent medical and physiological consequences in adult life.^2^^,^^3^^,^^5^ The distribution of the frequency of nutritional disorders, which has an infinite range, is widely used by healthcare professionals and researchers to classify the nutritional status of children around the world.^6^

The rates of nutritional disorders among children, both in developing and in developed countries, constantly change.^1^^,^^2^^,^^7^ For example, recent representative sampling studies in Brazil have shown a decrease in stunting, wasting and underweight and an increase in overweight among children under five years of age.^8^^,^^9^ These findings characterize a nutritional transition that has occurred over recent decades in many places throughout the world. This transition has been a result from globalization relating to the characteristics of family lifestyle and food consumption at all socioeconomic levels.^10^ However, this change has not consistently improved children’s health, and this reflects the challenges faced by social and healthcare systems in aiming to meet the global commitment defined in the United Nations’ health-related Millennium Development Goals (MDGs).^7^^,^^11^

Understanding the risk factors involved in malnutrition and overnutrition has helped healthcare professionals identify children who are vulnerable to nutritional disorders, thus determining the priorities for prevention and control action plans. Although the multiple determinants of nutritional disorders are interlinked and form a system of influences,^4^ the results relating to malnutrition and overnutrition have usually been reported separately in the literature. In addition, studies have not identified risk factors that could lead to either type of nutritional disorder (malnutrition or overnutrition) and have not provided guidance for preventing all the concomitant nutritional disorders.^4^^,^^12^

Educational institutions also have a responsibility in relation to concern about nutritional disorders during the first years of life. The numbers of children attending nurseries in day-care centers in Brazil, as an initial and alternative educational environment for the children of working women, has been increasing. Currently, more than 6.7 million Brazilian children are enrolled in day-care centers and kindergartens.^13^

To gain insight into this setting and inform healthcare strategies and policies that might more effectively protect children in a changing world, we conducted a study on children attending day-care centers in the city of São Paulo, in order to estimate the proportion of children with nutritional disorders and identify individual and combined risk factors for malnutrition and overnutrition.

## OBJECTIVE

The objective of this study was to estimate the frequency of nutritional disorders and to determine the risk factors for these diseases among children at 13 nurseries in São Paulo.

## METHODS

The present study was part of “Projeto CrechEficiente” (Efficient Nursery Project), and the methods have been reported elsewhere.^14^

In brief, we used data from two surveys (2004 and 2007) on infant populations attending nurseries (ages 0 - 2 years) that were located in public day-care centers (directly administrated by the city) and philanthropic day-care centers (indirectly administrated, through philanthropic institutions) that abided by admission rules guaranteeing care for low-income families.

Survey 1 was undertaken in 54 day-care centers located in the central region of the city of São Paulo, Brazil, and survey 2 was conducted in 36 day-care centers in the subdistrict of Santo Amaro in the same city. The managers of the day-care centers were contacted by telephone to identify which ones were eligible. Of these, 47 day-care centers were excluded because they did not have a nursery, 4 were excluded because they refused to participate and 8 were excluded because they had been involved in a previous healthcare study. This resulted in 13 and 18 day-care centers included in surveys 1 and 2, respectively.

The 31 day-care centers were visited by the project’s field staff, and a questionnaire asking for information about the school’s operations, the characteristics of their human resources and the numbers and ages of the children attending was completed. Afterwards, these day-care centers were ranked according to the characteristics of interest for the project.

The following criteria were prioritized in order of decreasing value: number of children in the nursery, number of nursery teachers, safety of the area for the researchers (because some day-care centers were located in areas with high rates of violence) and ease of transportation and access to the premises. Accordingly, five day-care centers from survey 1 and eight from survey 2 were selected for the final stage of data collection, thus totaling 13 day-care centers.

The initial population of the 13 day-care centers selected consisted of 498 children, aged 4 to 29 months, who were enrolled at and were regularly attending the nurseries. The following children were excluded: four children who were not present during the field activities, five children who had acute diseases at the time of the surveys, five disabled children with chronic conditions (two with Down’s syndrome, one with another genetic syndrome and two with cerebral palsy) and two children whose guardians refused to participate or did not sign the informed consent statement.

Seventeen other children were excluded only from the multivariate analysis because some of the data for the variables were missing. Therefore, 482 children were selected and included in the univariate analysis, and 465 children were included in the multivariate analysis, with sample losses of 3.2% and 6.6%, respectively.

A structured and pre-encoded questionnaire was used to collect individual data on the children, including demographic, clinical, epidemiological, socioeconomic and environmental variables. To ensure uniformity in the fieldwork procedures carried out by the interviewers, a manual of norms and definitions was created.^15^

Data collection was conducted in the day-care centers by interviewing the mothers or guardians, performing anthropometry and collecting blood samples from the children by means of digital puncture. All procedures were standardized and tested at the pretest stage of the project by an interdisciplinary field team consisting of postgraduate students.

The completed questionnaires were evaluated for their internal consistency before data entry. The information was transcribed into databases using double entry and subsequent validation to correct errors.

The children were weighed on a digital pediatric scale (BP Baby model, Filizola) and their lengths/heights were measured using an anthropometric ruler with a movable cursor. Both of these were made by Brazilian manufacturers with international quality certification. The anthropometric procedures used were in accordance with international recommendations.^6^ Z-scores were used to express nutritional status, in conformity with the Multicentre Growth Reference Study (MGRS) standards for age and sex, as recommended by the World Health Organization (WHO) in 2006.^6^

Stunting was defined as a situation with length/height-for-age z-scores < -2 SD; wasting as weight-for-height < -2 SD; underweight as weight-for-age < -2 SD; and overweight as weight-for-height or body mass index-for-age > 2 SD.^3^^,^^5^ To measure hemoglobin (Hb) levels, a portable Hb photometer (HemoCue hemoglobin photometer) was used.^16^

The outcomes investigated were malnutrition (stunting, wasting or underweight), overnutrition (overweight) and nutritional disorders (stunting, wasting, underweight or overweight), based on the WHO 2006 standards.^3^^,^^6^

To investigate the associations between categorical variables, the chi-square (x^2^) test was used.^17^ The cutoff points for the dichotomous variables were based on the official recommended values or on the average value of the variables in the study sample.^6^^,^^15^

To adjust for confounding factors, multivariate analysis was performed using the “stepwise forward” technique. The selection criterion for the explanatory variables for inclusion in the three logistic models was presence of an association with the outcomes at the level of P < 0.20.^18^

In addition to inclusion of the variables with statistically significant associations in relation to the logistic model outcomes (maximum level of P = 0.05), we also included the following control variables: age, hemoglobin level and *per capita* family income.

Children who had not received the vaccine doses expected for their age, in accordance with the Brazilian basic vaccination calendar, were considered to have incomplete vaccination. We also created the variable “neonatal risk”, to include children who were premature (gestational age less than 37 weeks), had low birth weight (less than 2.5 kg) and were kept in hospital at the neonatal stage for reasons relating to these conditions.

Finally, to verify the fit of the logistic regression models, we used the Hosmer-Lemeshow goodness-of-fit test.^18^ The statistical package used was the Stata software, version 11.0 (StataCorp, College Station, TX, USA).

Children identified as having a nutritional disorder, including anemia, were referred for outpatient care at the nutritional specialty unit in the Department of Pediatrics of the same university. Children with incomplete vaccinations were referred to healthcare centers for appropriate immunizations. This study was approved by the university’s research ethics committee.

This study was conducted in accordance with the guidelines laid down in the Declaration of Helsinki, and all procedures involving human subjects were approved by the research ethics committee. Written informed consent was obtained from the parents of all of the children.

## RESULTS

Out of the 482 children, 248 (51.4%) were boys and 234 (48.6%) were girls. Sixty-two percent of them were enrolled at philanthropic day-care nurseries, and 38% were enrolled at public day-care nurseries. The average age was 17.2 months (95% confidence interval, CI, 16.7 - 17.8), and the average duration of exclusive breastfeeding was 2.1 months (95% CI: 1.9 - 2.3).

The prevalences of prematurity, low birth weight and neonatal hospitalization were 10.8% (95% CI: 8.0 - 13.6), 9.8% (95% CI: 7.1 - 12.5) and 4.8% (95% CI: 2.9 - 6.7), respectively. The proportion of children who presented neonatal risk was 3.0% (95% CI: 1.4 - 4.5); and 6.2% (95% CI: 4.1 - 8.4) of the children had incomplete vaccination.

**Figure 1.**
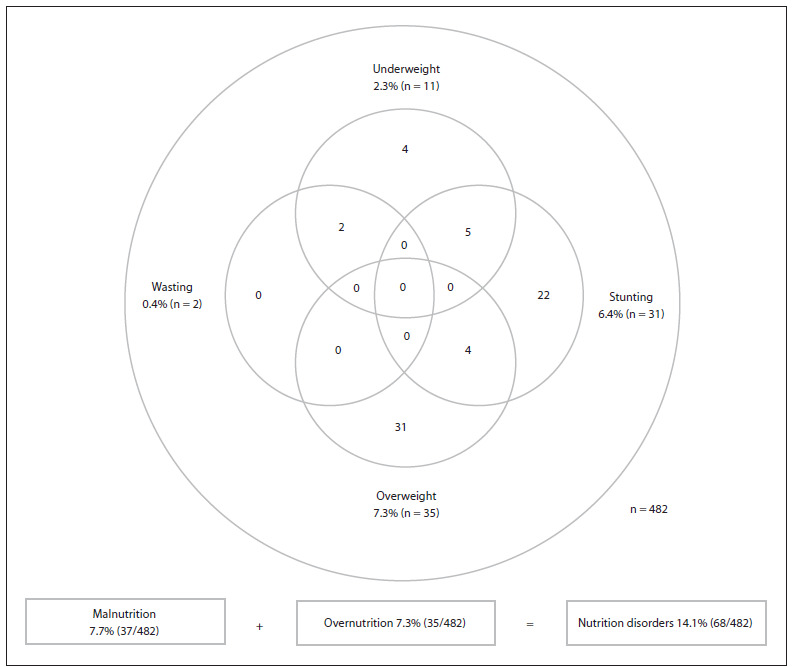
Prevalence and numerical intersection of groups of children with nutritional disorders (stunting, wasting, underweight or overweight) in public and philanthropic day-care nurseries in the municipality of São Paulo, from two surveys (2004 and 2007).

We found that 7.7% (95% CI: 5.5 - 10.5) of the children were malnourished, 7.3% (95% CI: 5.2 - 10.0) were overnourished and 14.1% (95% CI: 11.2 - 17.6) had at least one nutritional disorder. All children who presented wasting (n = 2; 0.4%) were also underweight (n = 11; 2.3%), and the most frequent type of malnutrition was stunting (n = 31; 6.4%). Figure 1 presents the prevalence and overlapping nutritional disorders (stunting, wasting, underweight and/or overweight).

Table 1 presents the unadjusted and adjusted odds ratios (ORs) and 95% CI for the risk factors for malnutrition. Among the socioeconomic variables, *per capita* family income of less than half the minimum monthly wage (MW) was the first one identified to form part of the logistic model. Three other variables indicating individual child processes, “incomplete vaccination status”, “neonatal risk” and “male sex”, were also selected to form part of the final model. Therefore, children who had these characteristics were 2.2, 3.4, 8.1 and 3.7 times more likely to be malnourished, respectively.

**Table 1.**
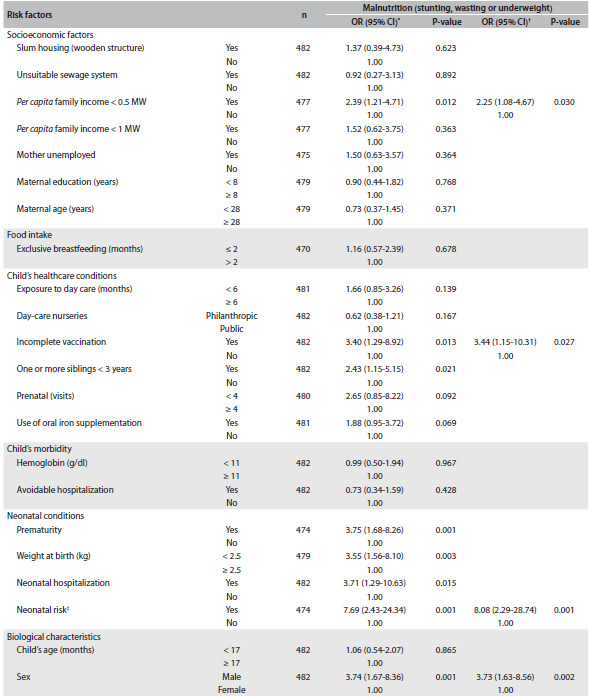
Unadjusted and adjusted odds ratios (ORs) with their respective confidence intervals (95% CIs) for the risk factors for malnutrition (stunting, wasting or underweight) among children in public and private day-care nurseries in the municipality of São Paulo, from two surveys (2004 and 2007)

CI = confidence interval; MW = minimum monthly wage; ^*^Unadjusted OR; ^†^OR adjusted for the variables making up the final multiple logistic model (P < 0.001); ^‡^low birth weight (less than 2.5 kg) + prematurity (gestational age less than 37 weeks) + neonatal hospitalization; control variables: age and hemoglobin level.

Table 2 presents the unadjusted and adjusted ORs and 95% CIs for the factors associated with overnutrition. The multiple logistic model showed that children who had shorter periods of exclusive breastfeeding (less than two months) and those who were male were 3.0 and 2.2 times more likely to be overnourished, respectively. On the other hand, children of families with lower *per capita* income were at lower risk of developing overnutrition (OR: 0.4). These three variables were independently associated with this outcome.

**Table 2.**
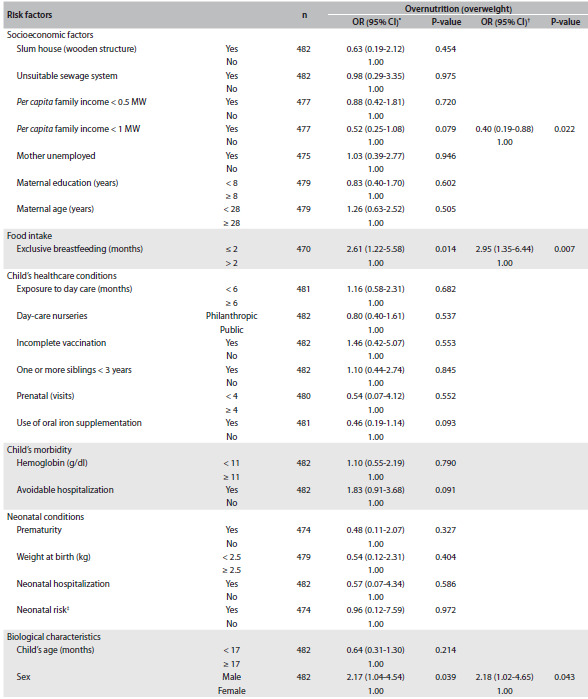
Unadjusted and adjusted odds ratios (ORs) with their respective confidence intervals (95% CIs) for the risk factors for overnutrition (overweight) among children in public and private day-care nurseries in the municipality of São Paulo, from two surveys (2004 and 2007)

CI = confidence interval; MW = minimum monthly wage; ^*^Unadjusted OR; ^†^OR adjusted for the variables making up the final multiple logistic model (P = 0.003); ^‡^low birth weight (less than 2.5 kg) + prematurity (gestational age less than 37 weeks) + neonatal hospitalization; control variables: age and hemoglobin level.

Table 3 presents the unadjusted and adjusted ORs and 95% CIs for the factors associated with the set of nutritional disorders. Children who presented neonatal risk (OR: 3.4), had incomplete vaccinations (OR: 3.1) or were male (OR: 2.7) presented independent associations with higher risk of nutritional disorders (stunting, wasting, underweight or overweight).

**Table 3.**
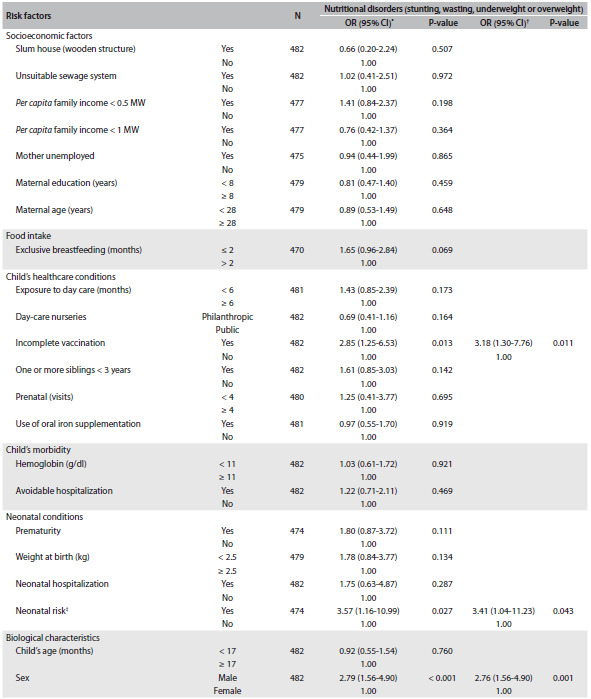
Unadjusted and adjusted odds ratios (ORs) with their respective confidence intervals (95% CIs) for the risk factors for nutritional disorders (stunting, wasting, underweight or overweight) among children in public and private day-care nurseries in the municipality of São Paulo, from two surveys (2004 and 2007)

CI = confidence interval; MW = minimum monthly wage; ^*^Unadjusted OR; ^†^OR adjusted for the variables making up the final multiple logistic model (P < 0.001); ^‡^low birth weight (less than 2.5 kg) + prematurity (gestational age less than 37 weeks) + neonatal hospitalization; control variables: age, hemoglobin level and *per capita* family income.

In the three logistic regressions, other variables met the selection criteria for inclusion in the logistic model (P < 0.20). However, these were not kept in the analysis because they lost their statistical significance when included in the final model.

## DISCUSSION

Overweight and stunting were the most common nutritional disorders in children in day-care centers in Brazil. Four factors were independently and significantly associated with malnutrition: low *per capita* family income, neonatal risk, incomplete vaccination status and male sex. Meanwhile, three factors were associated with overnutrition: high *per capita* family income, short exclusive breastfeeding and male sex. Furthermore, three factors were associated with the set of nutritional disorders: neonatal risk, incomplete vaccination status and male sex.

Childhood overnutrition is a complex condition that is influenced by our evolutionary genetic legacy interacting with a technologically advanced and consumerist society, thereby generating multiple associated risk factors.^12^ These determinants appear to be closely related to perinatal conditions, early weaning and high purchasing power, and they lead to excess energy intake and consumption of manufactured food.^12^^,^^19^

Similarly, child malnutrition is a process involving many risk factors relating to lower economic resources (poverty), lower food availability and lower maternal education, which leads to lack of energy and protein intake.^4^

The risk factors identified in the present study were independent of the funding source of the day-care centers (public or philanthropic), thus indicating that they are important for children in low *per capita* income families attending both day-care center systems. However, it is worth noting that even though the study was conducted using rigorous data collection and analysis, the selection of day-care centers was based on ease/convenience, giving priority to centers with a large number of children and to those that were located in poor but safe areas. Additionally, it is possible that the numbers of subjects and day-care centers used in this study were not large enough to provide a broad perspective of the risk of nutritional disorders among children attending this level of schooling. Consequently, it may not be possible to generalize these results to all Brazilian day-care centers, and the external validity and possibility of generalizing these results to children who are not in day care or to children attending day-care nurseries that function in other contexts must be considered only with caution.

Since the analysis was conducted on an existing dataset, we were limited to the use of variables that were collected for the “Projeto CrechEficiente”. For instance, our study did not investigate maternal state of health or the food consumption characteristics (quality and quantity of nutrient intake), which are known to be associated with both overnutrition and malnutrition in both developed and developing countries.^3^^,^^4^^,^^12^

Consistent with our study, other researchers have found higher rates of overweight and stunting than of wasting and underweight in Brazil.^9^^,^^11^^,^^19^^,^^20^ However, the prevalence of malnutrition was similar to that of overnutrition among children attending day-care nurseries. This result suggests that the Brazilian health and education systems are unable to meet their goals of keeping the anthropometric indices of children attending day-care centers close to the average for the general population of the same age, and thus these children are not protected from nutritional disorders.

The only factor present in all three final models was male sex, thus indicating that boys are more likely to develop overnutrition, malnutrition and the set of nutritional disorders. Some studies reported that there was no relationship between sex and child nutrition indices.^21^ However, another study reported that nutritional states were worse among males,^19^ while others reported that females are nutritionally disadvantaged.^22^ Similarly, some researchers have not found associations between being overweight and sex,^12^ while others reported that male sex was a determinant of childhood obesity.^23^ This controversy appears to be the result of sex only being one component of a more complex system of linked influences. Specifically, there is evidence that the relationship between sex and nutrition is modulated by a variety of factors, including cultural values, birth order, number of male and female children in the family, household and the day-care centers’ decisions regarding allocation of food resources.^4^^,^^24^

Being up-to-date with vaccinations has been reported to be negatively associated with malnutrition.^25^ This probably reflects a common determinant of parental education/knowledge and accessibility to primary healthcare services, thus suggesting that a lack of preventive healthcare during the first years of life can result in infant malnutrition. However, the literature has not shown any association between being overweight and incomplete vaccination status.^12^ Consistently, in the present study, vaccination status was associated with malnutrition, but not with being overweight. Nonetheless, incomplete vaccination status had the largest impact on the set of nutritional disorders.

Children with a history of being weaned from breastfeeding before two months of age showed a higher risk of overnutrition, which was consistent with another study.^26^ In contrast, two reviews concluded that breastfeeding is unlikely to be protective of early childhood obesity because associations might be explained largely by residual confounding and/or publication bias.^12^^,^^27^ However, short exclusive breastfeeding was not associated with malnutrition, as reported in many other studies.^4^^,^^25^

Low *per capita* family income has also been reported to be associated with malnutrition^4^^,^^19^^,^^20^ and overnutrition.^8^^,^^12^^,^^19^ This is likely to be indicative of a parent’s ability to appropriately care for their children by providing more and adequate feeding.

Despite the fact that we analyzed children from low-income families and not from the general community, our results showed that children of families with lower *per capita* income had a higher risk of malnutrition, whereas those with higher *per capita* income had increased risk of overweight, similar to the results from other studies conducted in various Brazilian regions.^8^^,^^20^ As expected, the association between the nutritional disorders was not as strong. This finding emphasizes the importance of appropriate use of available socioeconomic resources and of improving socioeconomic conditions in the poorest groups, so as to prevent and control these nutrition imbalances.

Nevertheless, the traditional explanation that child nutritional deficiencies are simply due to food scarcity or a lack of family economic resources has been increasingly questioned by researchers.^4^ Furthermore, the roles of three caregiver/parent characteristics (education, intelligence and good mental health) have been considered to be important determinants linked to characteristics such as culture, maternal input into family economic decisions and social support networks.^4^

In contrast to some other studies,^4^^,^^8^^,^^19^^,^^22^ the present investigation did not identify any statistically significant association between malnutrition or overnutrition (or a combination of them) and maternal employment, age or education. This may have resulted from the lack of diversity of these variables in the study population.

Infants born prematurely, who were of low birth weight or who were hospitalized during the neonatal period were found to be more likely to acquire nutritional disorders in the first months of life.^28^ However, we did not find any association between neonatal risks and being overweight, although neonatal risk was associated with malnutrition and with the set of nutritional disorders, even after controlling for age, hemoglobin level and *per capita* family income.

These findings gain greater validity through inclusion of other factors that can influence nutritional status in multivariate analysis, thereby providing a broad perspective for events that are triggered by multiple risk factors.^18^ As such, the effects were shown to be statistically significant when controlling for the other variables in the multifactorial model, including hemoglobin level, as an essential indicator of iron status, and adequacy of food intake.

Governments in developing countries have considered strategies, such as visits to vulnerable households and integrated care across emergency and primary care services, to be methods for improving the nutritional status of children.^11^ However, it is also important to recognize the potential for day-care nurseries to improve the health of children under their care during weekdays and to ensure adequate nutrition for them. Nurseries are considered to be spaces for implementing programs to control and prevent infant sociobiological vulnerability to parents. Managers and healthcare professionals believe that healthcare actions at day-care centers and health education programs directed towards children’s caregivers can improve a child’s health.^30^

Furthermore, this study highlights that there are risk factors associated with the set of nutritional disorders, which is a new perspective for nutritional strategies. Identification of the prevalence of overnutrition and malnutrition, individually and in combination, along with the associated factors, suggests that opportunities to keep children nutritionally healthy, as demonstrated through anthropometric measurements, are being missed.

Finally, we emphasize the need for comprehensive health and nutritional interventions in day-care centers that address nutritional disorders and also the importance of keeping children’s anthropometric patterns close to the average for the population.

We recommend that future studies should use qualitative and quantitative methods to study the determinants of nutritional disorders based on a comprehensive sample of children attending day-care centers, in order to generate further epidemiological evidence to inform health and educational interventions for preventing nutritional disorders.

## CONCLUSION

Based on the results from this study, we conclude that nutritional disorders remain present among children attending nurseries in São Paulo. Knowledge of the risk factors for overnutrition, malnutrition and the set of nutritional disorders can potentially help healthcare professionals develop strategies to prevent and control these nutrition imbalances. Actions to enhance the nutritional status of children should focus on boys, children who were exclusively breastfed for less for than two months and those without up-to-date vaccinations. Identification of risk factors for a set of nutritional disorders seems useful as a new approach for supporting health promotion actions.
